# Comparison of the Tele‐Oxford Cognitive Screen to a neuropsychological battery in chronic stroke survivors

**DOI:** 10.1111/jnp.70021

**Published:** 2025-11-15

**Authors:** Ye Wo, Nele Demeyere, Sam S. Webb

**Affiliations:** ^1^ Department of Experimental Psychology University of Oxford Oxford UK; ^2^ Department of Psychiatry University of Oxford Oxford UK; ^3^ Nuffield Department of Clinical Neurosciences University of Oxford Oxford UK

**Keywords:** cognitive impairment, cognitive screening, neurorehabilitation, stroke, validation

## Abstract

Following an increased need for remote cognitive screening solutions, we aimed to investigate the construct validity and determine initial sensitivity/specificity estimates of the Tele‐OCS, a stroke‐specific remotely administered cognitive screening tool. To this end, a secondary data analysis is presented from 98 stroke survivors from the OX‐CHRONIC longitudinal study (average 4.5 years. post‐stroke). Convergent validity was examined for overall Tele‐OCS performance against MoCA total score, and separately for each of the subtasks against matched neuropsychological tasks. Divergent validity was examined against different neuropsychological tests and presumed to be unrelated self‐reported anxiety, as measured with HADS‐A. Overall, we found that the Tele‐OCS subtasks had good convergent/divergent validity. All subtasks also showed excellent specificity (min 80%), and whilst the cancellation task also showed good sensitivity (80%), all other subtasks came at a cost of lower sensitivity, compared to a more sensitive neuropsychological assessment. The Tele‐OCS provides a brief, remote, first‐line cognitive screening tool that reliably detects cognitive changes where these are clearly present, specifically and validly measuring distinct cognitive domains, which contrasts with a domain‐general cognitive screening approach.

**Practitioner points**

**Clinical implications**

- The Tele‐OCS offers a validated, stroke‐specific remote cognitive screening tool for chronic stroke, providing clinicians with an alternative modality when in‐person assessment is not possible.
- Subtasks reliably capture distinct cognitive domains, supporting domain‐specific clinical decision‐making, rather than relying solely on global screening measures.
- The high specificity of the Tele‐OCS indicates that identified impairments are likely genuine, helping reduce false positives and unnecessary referrals.
- Remote administration increases accessibility, particularly for patients in rural or resource‐limited settings, facilitating continuity of care.

**Cautions and limitations**

- While specificity was high, sensitivity was limited for half of subtasks, suggesting that subtle cognitive impairments may be missed compared to a full neuropsychological battery.
- The study sample comprised chronic stroke survivors, meaning findings may not generalize directly to acute populations where impairments are more prevalent.
- Some subtasks demonstrated ceiling effects, restricting variability and limiting correlations with comparison measures.
- As this study used secondary data, not all Tele‐OCS subtasks could be validated against gold‐standard neuropsychological comparisons.

Stroke is a prominent health concern worldwide, affecting an additional 100,000 individuals each year in the United Kingdom (King et al., 2020). Among the challenges faced by stroke survivors, cognitive impairment emerges as a prominent issue that frequently accompanies stroke‐induced disability (Pendlebury et al., 2019). Screening for cognitive impairment after stroke is now standard practice, recommended by national and international clinical guidelines (e.g., Quinn et al., 2021).

The Oxford Cognitive Screen (OCS) (Demeyere, Riddoch, Slavkova, Bickerton, & Humphreys, 2015) was developed as an in‐person stroke‐specific cognitive screen on pen and paper for use in acute settings. In the wake of COVID‐19, the pandemic significantly increased the demand and accelerated the adoption of online administered cognitive tests, as healthcare professionals swiftly shifted to telephone or video conferencing modalities for assessments (Pucci et al., 2024). The OCS was formally converted for remote administration by the development team, following research showing that make‐shift remote versions of the OCS were being used in the United Kingdom (Webb et al., 2021). We intend for the Tele‐OCS to be used in subacute and chronic stages post‐stroke. Preliminary research provided evidence of validity for the remote administration of the Tele‐OCS, examining equivalency to the paper and pen administered version (Webb et al., 2023), but not its construct validity or sensitivity/specificity. The traditional paper OCS has previously been compared to a construct and format matched neuropsychological battery, where moderate to large correlations were observed between OCS subtests and comparison subtests (min *r* = .31, max = 70, average = .55), but no such comparison has been made with the Tele‐OCS to date.

Here, we leverage secondary data to examine the construct validity for the tele‐OCS. The OX‐CHRONIC study investigated long‐term psychological outcomes in a longitudinal stroke cohort (Kusec et al., 2023), who were assessed on the Tele‐OCS remotely on paper booklets alongside a battery of neuropsychological assessments as well as the brief MoCA screen (Nasreddine et al., 2005).

## METHODS

The study obtained ethical approval from the NRES Ethics Committee (REC Reference: 19/SC/0520). All participants received informed consent. Full details of OX‐CHRONIC are available in the protocol (Demeyere et al., 2021) and overall report of OX‐CHRONIC (Kusec et al., 2023).

### Participants

Participants were at least 2 years post stroke (median 3.84 years). While 105 stroke survivors took part, only 98 completed the cognitive assessments in full and are included here for analysis (average age 73.18 (*SD* = 12.39), average years of education 13.98 (*SD* = 3.56), 42% female, 83% ischaemic stroke, 67% first ever stroke, average acute stroke severity as measured by NIHSS = 7.27 (*SD* = 6.27)). Details on the prevalence of cognitive and mood scores for this sample are extensively described elsewhere (Kusec et al., 2023).

### Cognitive measures

The Tele‐OCS is a remotely administered pen‐and‐paper version of the Oxford Cognitive Screen (OCS) version A (Demeyere, Riddoch, Slavkova, Bickerton, & Humphreys, 2015), to facilitate remote screening for stroke‐specific cognitive impairments (Webb et al., 2023) and is available through Oxford University Innovations licensing at no cost for publicly funded clinical and research use. Continuous performance scores are generated for each subtask, and impairment levels are determined based on structured cutoffs from the traditional OCS (Demeyere, Riddoch, Slavkova, Bickerton, & Humphreys, 2015), as we did not include neurologically healthy controls.

Neuropsychological comparison tasks included the Star Cancellation Test (Wilson et al., 1987), Cookie Theft Task (Borod et al., 1980), DKEFS Letter and Category Fluency and DKEFS Trail Making Test A and B (Delis et al., 2001), Hayling Sentence Completion Test (Burgess & Shallice, 1997), OCS‐Plus Trail Making Test (Demeyere et al., 2021), WMS‐III Digit Span Forwards and Backwards and Logical Memory Test I and II (Wechsler, 1997), Picture Memory Test for memory the Boston Naming Test (Kaplan et al., 1983) and the Rey‐Osterrieth Complex Figure Copy Test (Osterrieth, 1944). In addition, Tele‐OCS is compared to a brief cognitive screen in the Montreal Cognitive Assessment (MoCA) (Nasreddine et al., 2005). Requirements (i.e., licensing etc) for the use of the tests were satisfied.

Emotional distress was measured using the Hospital Anxiety and Depression Scale (Zigmond & Snaith, 1983).

The order of administration was fixed, with all participants completing the Tele‐OCS first, followed by a set of neuropsychology tests, in order to minimize potential order effects and standardize the procedure, ensuring that performance on the Tele‐OCS was not influenced by prior exposure to the neuropsychology battery.

### Tele‐OCS administration

Upon obtaining their consent to participate, the Tele‐OCS pack was dispatched to the participants via mail at least one week prior to their scheduled session. Participants were explicitly instructed to refrain from opening the envelope until their designated appointment. Depending on the individual's preference, the administration of the Tele‐OCS and neuropsychological tests was conducted either via phone or videoconferencing. Once the session was completed, participants were kindly requested to return the testing packs through mail, utilizing the pre‐paid envelopes provided by the laboratory. The administration of each task followed established guidelines and instructions as outlined in the published protocol (Demeyere et al., 2021). Comments on any issues encountered were recorded by examiners.

### Data analysis

Data analysis was conducted using R version 2024.12.0 + 467 (R Core Team, 2022) in RStudio version 4.2.2 (Posit team, 2024). The analysis scripts, allowing for the recreation of the manuscript, are publicly available under the CC‐BY 4.0 licence (https://doi.org/10.17605/OSF.IO/94YMT). Data were obtained from the open‐access Ox‐CHRONIC data set (https://osf.io/y2mev/files/osfstorage) (Kusec et al., 2023).

We investigated overall convergent validity between the proportion of Tele‐OCS impairments (as a total score proxy, reflecting overall severity) and overall MoCA score. In addition, task‐specific convergent and divergent validity of the Tele‐OCS subtasks was compared to neuropsychological measures. All comparisons use Kendall's Tau correlation analyses, with benchmarks where correlations greater than Pearson's *r* = .30 (Kendall Tau >.20) are considered as evidence for convergence (and below for divergence), similar to previous validations in cognitive screening (Demeyere et al., 2021; Webb et al., 2022).

For sensitivity/specificity estimates, we compared Tele‐OCS and neuropsychological battery subtask impairment classifications, based on published impairment cut‐offs (Kusec et al., 2023).

## RESULTS

### Overview

Performance scores indicated that 45.71% of the participants exhibited at least one subtask impairment on the Tele‐OCS screening. Neuropsychological assessment revealed that 59.05% of participants demonstrated at least one impairment. Regarding the MoCA scores, the average score was 23.56 (SD 4.16). Depending on the cut‐off used, 65.31% (<26, original cut‐off (Nasreddine et al., 2005)) or 30.61% (<23, the recommended cut‐off for stroke (Pendlebury et al., 2010)) of participants were classed as impaired on MoCA.

### Convergent and divergent validity

A robust moderate correlation was observed between the proportion of tasks impaired from the Tele‐OCS and the MoCA total score (*τ*(96) = −.40, *p* < .001).

Convergent and divergent correlations of the Tele‐OCS with corresponding neuropsychological tests are presented in Table 1.

**TABLE 1:** Convergent and divergent correlations between Tele‐OCS tests and neuropsychological validation tests.

Tele‐OCS subtask	Convergent test	Convergent correlation	Divergent test	Divergent correlation
Picture naming	BNT correct no cue	*τ*(96) = .29, *p* = .002*	Rey Complex Figure Copy: Copy	*τ*(95) = .14, *p* = .094
	MoCA naming	*τ*(96) = .12, *p* = .219	HADS anxiety	*τ*(90) = .00, *p* = .958
Orientation	MoCA orientation	*τ*(96) = .4, *p* < .001**	Rey Complex Figure Copy: Copy	*τ*(95) = .14, *p* = .102
			HADS anxiety	*τ*(90) = .04, *p* = .675
Number writing	MoCA clock number writing	*τ*(94) = .08, *p* = .456	BNT correct no cue	*τ*(94) = .12, *p* = .19
			HADS anxiety	*τ*(88) = .01, *p* = .943
Number calculation	MoCA serial subtraction	*τ*(96) = .38, *p* < .001**	BNT correct no cue	*τ*(96) = .08, *p* = .413
			HADS anxiety	*τ*(90) = .11, *p* = .194
Sentence reading			Rey Complex Figure Copy: Copy	*τ*(96) = .08, *p* = .413
			HADS anxiety	*τ*(90) = 0.11, *p* = .194
Broken hearts total	BIT star total	*τ*(95) = .23, *p* = .005*	BNT correct no cue	*τ*(96) = .14, *p* = .082
			HADS anxiety	*τ*(90) = .13, *p* = .102
Broken hearts space asymmetry	BIT star laterality	*τ*(95) = .10, *p* = .218	BNT correct no cue	*τ*(96) = .11, *p* = .192
			HADS anxiety	*τ*(90) = .02, *p* = .802
Broken hearts object asymmetry		*τ*(95) = .13, *p* = .143	BNT correct no cue	*τ*(96) = .08, *p* = .368
			HADS anxiety	*τ*(90) = .15, *p* = .09
Free verbal recall	MoCA delayed recall	*τ*(96) = .45, *p* < .001**	Rey Complex Figure Copy: Copy	*τ*(95) = .07, *p* = .363
			HADS anxiety	*τ*(90) = .07, *p* = .37
Episodic recognition	Logical Memory Test delayed recall	*τ*(95) = .32, *p* < .001**	BNT correct no cue	*τ*(95) = .09, *p* = .313
	Picture memory test	*τ*(96) = .21, *p* = .018*	HADS anxiety	*τ*(90) = .1, *p* = .268
Trails mixed	MoCA trails	*τ*(96) = .37, *p* < .001**	BNT correct no cue	*τ*(96) = .13, *p* = .14
	Hayling Sentence Completion Test total	*τ*(94) = .22, *p* = .006*	HADS anxiety	*τ*(90) = 0.08, *p* = .31
	OCS‐Plus trails mixed	*τ*(95) = .41, *p* < .001**	BNT correct no cue	*τ*(96) = .13, *p* = .14
Trails exec	MoCA trails	*τ*(96) = .3, *p* = .001	BNT correct no cue	*τ*(96) = .07, *p* = .401
	Hayling Sentence Completion Test total	*τ*(94) = .2, *p* = .014*	HADS anxiety	*τ*(90) = .09, *p* = .252
	OCS‐Plus trails exec	*τ*(95) = .22, *p* = .008*	BNT correct no cue	*τ*(96) = .07, *p* = .401
Figure copy	Rey Complex Figure Copy: Copy	*τ*(95) = .47, *p* < .001**	BNT correct no cue	*τ*(96) = .09, *p* = .25
			HADS anxiety	*τ*(90) = .06, *p* = .42
Figure recall	Rey Complex Figure Copy: Recall	*τ*(94) = .55, *p* < .001**	BNT correct no cue	*τ*(96) = .11, *p* = .168
			HADS anxiety	*τ*(90) = 0.09, *p* = .247

*Note*: For the Tele‐OCS Semantics task, all participants achieved a full score of 3, resulting in a lack of variation in the scores, thereby preventing correlational analysis. Furthermore, the sentence reading and object neglect scores from the Tele‐OCS had no comparisons in the neuropsychological battery, as it was opportunity data and as such no associations could be computed.

Significance at <.05 is ‘*’ and <.001 is ‘**’.

### Sensitivity and specificity

Sensitivity and specificity analyses for each of the Tele‐OCS subtasks compared to a matched Neuropsychological reference task assessment are outlined in Table 2.

**TABLE 2:** Impairment classification of the Tele‐OCS compared to neuropsychological tasks.

Tele‐OCS subtask	Neuropsych. Test	True positive (%)	True negative (%)	False positive (%)	False negative (%)	Sensitivity (%)	Specificity (%)
Picture naming	BNT correct no cue	1.02	92.86	3.06	3.06	25.00	96.81
Broken hearts total	BIT star total	4.12	77.32	17.53	1.03	80.00	81.52
Broken hearts left space neglect	BIT star left neglect	2.06	87.63	7.22	3.09	40.00	92.39
Broken hearts right space neglect	BIT star right neglect	0.00	94.85	5.15	0.00	‐	94.85
Verbal recall (free recall and recognition)	Logical memory test immediate recall	0.00	91.75	5.15	3.09	0.00	94.68
	Logical memory test delayed recall	3.09	88.66	2.06	6.19	33.33	97.73
Episodic recognition	Logical memory test immediate recall	0.00	95.88	1.03	3.09	0.00	98.94
	Logical memory test delayed recall	0.00	89.69	1.03	9.28	0.00	98.86
	Picture memory test	1.02	73.47	0.00	25.51	3.85	100.00
Trails mixed	Hayling Sentence Completion Test total	6.25	63.54	5.21	25.00	20.00	92.42
	DKEFS trail b	1.03	84.54	10.31	4.12	20.00	89.13
	OCS‐Plus trails mixed	10.31	52.58	1.03	36.08	22.22	98.08
Trails exec	Hayling Sentence Completion Test total	6.25	63.54	5.21	25.00	20.00	92.42
	DKEFS trail b	0.00	83.51	11.34	5.15	0.00	88.04
	OCS‐Plus trails exec	2.06	75.26	9.28	13.40	13.33	89.02
Figure copy	Rey Complex Figure Copy: Copy	5.15	88.66	1.03	5.15	50.00	98.85
Figure recall	Rey Complex Figure Copy: Recall	3.13	88.54	3.13	5.21	37.50	96.59

Abbreviations: BIT, Behavioural Inattention Test; BNT, Boston Naming Test; DKEFS, Dellis–Kaplan Executive Function System; OCS‐Plus, Oxford Cognitive Screen‐Plus.

## DISCUSSION

Tele‐OCS as a whole and its subtasks demonstrated clear convergent validity. Overall, in this sample, a significant proportion of patients demonstrated cognitive impairment, with 45.71% showing an impairment on one of the Tele‐OCS subtasks. Overall performance, as measured by the proportion of Tele‐OCS tasks impaired correlated well with the MoCA total score, suggesting a degree of overall cognitive impairment severity is captured. All the Tele‐OCS subtasks showed good divergent validity. Similarly, good convergent validity was found for all, except for the number writing task (vs MoCA clock task) and the neglect asymmetry measure from the cancellation task. In terms of estimates of sensitivity and specificity, all the Tele‐OCS tasks showed excellent specificity (min 80%), though low sensitivity in all subtasks except for the cancellation task. We note this is in comparison to a more detailed (and lengthier) neuropsychological assessment test designed to maximize sensitivity. These results are in line with the established performance of the in‐person OCS, which has consistently demonstrated superior convergent validity to other commonly used screening tools such as the Montreal Cognitive Assessment (MoCA) (Nasreddine et al., 2005) and the Mini‐Mental State Examination (MMSE) (Cockrell & Folstein, 2002; Demeyere, Riddoch, Slavkova, Jones, et al., 2015; Mancuso et al., 2016; Robotham et al., 2020). Whilst overall good convergent and divergent validity was found, some correlations were relatively low in absolute terms. A clear limitation affecting the convergent and divergent validity measures is that some of the score ranges in the screening tests (Tele‐OCS and MoCA) were confined, leading to a lack of statistical variation necessary to yield larger correlation coefficients. Arguably, these subtasks were not designed with sufficient difficulty to elicit substantial score variations, which, at least in part, accounts for the diminished correlation strength. For example, the MoCA naming task only elicited scores of 2 or 3 and never 0 or 1; similar is true for the Tele‐OCS picture naming.

Whilst the Tele‐OCS subtasks were found to demonstrate high specificity, this came at the cost of sensitivity (in comparison to full neuropsychological assessment). On examination, the low sensitivity was mostly due to low rates of impairment in individual subtasks in the Tele‐OCS in this chronic stroke sample. We also previously found similar low impairment classifications in an independent chronic stroke sample comparing in‐person to remote testing (Webb et al., 2023). These findings suggest that the tele‐OCS is most valuable as a confirmatory tool, providing strong evidence of no severe impairment but limited ability to exclude more subtle deficits. Clinically, this supports a two‐stage screening process in which a broader, more sensitive screener is used first, with the tele‐OCS applied to confirm significant impairment where suspected. This approach aligns with the principles of the Mental Capacity Act (2025), prioritizing avoidance of false positive classifications while maintaining efficiency in remote assessment.

We note that the OCS (and tele‐OCS) was designed as a first‐line screening tool for use in acute stroke settings, which sees a much higher prevalence of stroke‐specific impairments, such as visuospatial neglect, expressive aphasia, etc. It is clear that many of these acute deficits recover over time (Milosevich et al., 2024). When choosing a cognitive screen, it is important to consider what the goal of the screening is. For example, one may want to determine whether pre‐existing decline (prior to the stroke) was already present and here an informant report such as IQcode (Jorm & Jacomb, 1989) may be most suitable. The OCS and Tele‐OCS were designed to detect new domain‐specific deficits following a new stroke. However, if the goal is to detect global (domain‐general) cognitive changes in a brief screen, use of MoCA or the OCS‐Plus could be recommended instead. OCS‐Plus has shown promise in demonstrating excellent alignment with neuropsychological testing (Webb et al., 2022), and higher sensitivity than MoCA (Roberts et al. 2024). A compromise solution, combining the domain‐specific nature of OCS and the sensitive and domain‐general nature of OCS‐Plus for a community‐dwelling stroke population was recently developed in the Mini‐OCS (Webb et al., 2025), showing excellent promise for a balanced brief screen specific to chronic stroke. It's also important to note that our study's focus is on a chronic, stable population. Acute or subacute stroke patients are often in the hospital or undergoing inpatient rehabilitation, where in‐person assessment is both feasible and necessary. The primary value of the tele‐OCS lies in providing a convenient and accessible long‐term management tool for a community‐dwelling chronic population, and the findings from this sample may not generalize to acute or subacute stroke populations.

There are several directions that future research can take to further validate and explore the clinical implications of the Tele‐OCS. Most notably, a validation study with an acute, and more severely cognitively impacted stroke cohort would enhance the generalizability of the Tele‐OCS and provide more reliable estimates of sensitivity and specificity. One sidenote, however, is that this particular group will be more challenging to recruit for research and may be unable to complete detailed and lengthy neuropsychological testing if these are used as the reference standard.

While our study validates the Tele‐OCS's ability to effectively capture cognitive impairment levels in a stroke survivor cohort, it does not provide new normative data (e.g., specific for its remote administration). Our preliminary data on the Tele‐OCS in chronic stroke in comparison to in‐person administration, strongly suggests the existing normative data may apply to the remote version (Webb et al., 2023). Indeed, the OCS as a test appears very stable across cultures and formats when considering impairment cut offs (Robotham et al., 2020). We acknowledge that an obvious crucial next step for future research is to validate these existing cutoffs specifically for the remotely administered version through the collection of data from a large and diverse sample of participants.

Finally, remotely administered versions of the OCS‐plus and Mini‐OCS may, in future, provide sensitive domain‐general screening suitable for stroke. Research into self‐administered versions further carries potential (though also drawbacks). For now, no other stroke‐specific alternative exists for remote cognitive screening.

## CONCLUSIONS

The present study provides substantial evidence supporting the construct validity of the Tele‐OCS as a domain‐specific cognitive screen, with good convergent and divergent validity. The convergent analysis reveals strong correlations between most Tele‐OCS tasks and matched neuropsychological tests, demonstrating the ability of the Tele‐OCS to effectively capture cognitive impairment levels similar to established measures. The high specificity observed in the sensitivity and specificity analysis underscores its accuracy in identifying cognitive impairment that is present in stroke survivors. By demonstrating strong overall impairment detection and significant correlations with established cognitive tests, the Tele‐OCS showcases its effectiveness as a valuable addition to cognitive assessment resources.

## AUTHOR CONTRIBUTIONS

**Ye Wo:** Methodology; investigation; formal analysis; writing – original draft; writing – review and editing. **Nele Demeyere:** Conceptualization; data curation; investigation; methodology; validation; supervision; funding acquisition; project administration; resources; writing – review and editing. **Sam S. Webb:** Conceptualization; methodology; formal analysis; data curation; validation; investigation; funding acquisition; writing – original draft; writing – review and editing; supervision.

## CONFLICT OF INTEREST STATEMENT

The author(s) declared the following potential conflicts of interest with respect to the research, authorship, and/or publication of this article: Nele Demeyere is a developer of the ‘Oxford Cognitive Screen’.

## Data Availability

The data that support the findings of this study are openly available in Open Science Framework at https://doi.org/10.17605/OSF.IO/94YMT and https://osf.io/y2mev/files/osfstorage.
